# Disarming oxidized lipids: AAV-delivered antibody neutralization as an emerging strategy against ALS

**DOI:** 10.1016/j.omta.2026.201879

**Published:** 2026-11-06

**Authors:** Sergi Verdés, Assumpció Bosch

**Affiliations:** 1 Institute of Neuroscience (INc), and Department of Biochemistry and Molecular Biology, Universitat Autònoma de Barcelona, Barcelona, Spain; 2 Unitat Mixta UAB-VHIR, Vall D’Hebron Institut de Recerca (VHIR), Barcelona, Spain; 3 Centro de Investigación Biomédica en Red Enfermedades Neurodegenerativas (CIBERNED), Instituto de Salud Carlos III, Madrid, Spain

## Main text

Amyotrophic lateral sclerosis (ALS) is a progressive and devastating neurodegenerative disease affecting motor neurons (MNs) and the neuromuscular axis. Despite decades of research on its molecular trigger and pathological mechanisms, and the search for effective disease-modifying therapies, both questions remain largely unknown. A common feature of ALS, present in 97% of cases, is the mislocalization, aggregation, and functional disruption of TAR DNA-binding protein 43 (TDP-43),^1^ yet the upstream trigger of this proteinopathy is poorly understood. Oxidized phosphatidylcholines (PC-OxPL) are danger-associated molecular patterns (DAMPs) generated by lipid peroxidation of membrane and lipoprotein phospholipids and can drive key features of neurodegeneration including inflammation, proteinopathy, and tissue injury.^2^^,^^3^^,^^4^ Their relevance to CNS disease is recent, with PC-OxPL detected in tissues from multiple sclerosis, frontotemporal dementia, Parkinson’s disease, and Alzheimer’s disease. Increasing evidence suggests that PC-OxPL are not merely markers of neurodegeneration but active mediators of the disease process.^4^^,^^5^

Recent data point to a specific role for PC-OxPL in ALS. Higher concentrations of phosphatidylcholines have been reported in the cerebrospinal fluid (CSF), and oxidized low-density lipoprotein (OxLDL) in the serum of ALS patients.^6^^,^^7^ In this issue of *Molecular Therapy Advances*, Gomes-Duarte et al.^8^ directly test this link by tracing PC-OxPL from its histopathological distribution in ALS tissue and CSF to its capacity to drive TDP-43 aggregation and induce an ALS-like phenotype across several disease models, and ultimately evaluate its neutralization as a therapeutic strategy.^9^

The authors show that PC-OxPL accumulate in cell bodies across several brain regions and the spinal cord from sporadic ALS patients (sALS), with selective enrichment in ChAT-positive spinal MNs, alongside a significant increase in several PC-OxPL species in the CSF. To further link PC-OxPL to ALS pathology, the authors exposed healthy iPSC-derived MNs (iMNs), alone and in co-culture with astrocytes, to the oxidized phospholipids PONPC and PAzPC, or their non-oxidized precursor PSPC. While PAzPC caused rapid cell death, PONPC induced clear TDP-43 aggregation without changes in phosphorylation or mitochondrial phenotype, suggesting a non-canonical form of TDP-43 pathology. NanoString transcriptomics of PONPC-treated iMNs identified extensive neuropathology and neuroinflammation-related transcriptomal changes, contrasting with bulk RNA-seq, which predominantly identified alterations in lipid metabolism, sterol/cholesterol biosynthesis, and SREBP-dependent pathways. This divergence suggests that the NanoString panel captures a transcriptional signature more closely associated with the acute insult and direct injury, whereas bulk RNA-seq may better reflect the progressive metabolic remodeling. The curated NanoString panel may therefore be biased toward acute stress-response genes rather than markers of chronic, disease-relevant neurodegeneration. Although such discrepancies between platforms are common, the authors do not establish which approach better captures the biology most relevant to ALS pathogenesis.

Among the most differentially expressed genes identified by NanoString after PONPC exposure, the authors report *APOE*, a major Alzheimer’s disease risk factor previously linked to impaired neuronal PC-OxPL clearance.^4^ Although *APOE* was not significant in any bulk RNA-seq comparison, protein analysis supported an ApoE response, with increased ApoE-positive cell counts and immunostaining in PONPC-exposed iMNs. Moreover, ApoE particles were the main PC-OxPL carriers in CSF, and it was increased in the CSF and spinal cord of ALS patients.

Having established PC-OxPL accumulation and pathogenic effects in ALS, the authors explored its therapeutic neutralization. Natural IgM antibodies recognize oxidized, but not native, phospholipids, but are not known to be present in the CNS.^3^ Based on this concept, in a previous study, Gomes-Duarte et al.^9^ developed PC-OxPL-VecTab®, an AAV5.2 vector encoding a secreted scFv designed to neutralize PC-OxPL in the CNS. In SOD1^G93A^ iMNs, which showed increased PC-OxPL and broad transcriptional dysregulation, PC-OxPL-VecTab® partially normalized the transcriptomic changes and nearly abolished PC-OxPL-induced axonal damage. *In vivo*, intrathecal administration in SOD1^G93A^ mice achieved robust biodistribution and reduced most plasma PC-OxPL species. The proposed mechanisms involve either masking PC-OxPL to prevent receptor engagement or promoting its clearance.

In a study published in the previous issue of *Molecular Therapy Advances*, the same construct was delivered to WT iMNs, prior to PONPC exposure. AAV5.2-PC-OxPL-VecTab normalized about 40% of PONPC-driven transcriptional activation and prevented TDP-43 aggregation.^8^ A logical next step would be to determine whether reduced aggregation translates into restoration of TDP-43 function. Functional readouts such as cryptic splicing of canonical TDP-43 targets (e.g., STMN2 and UNC13A) could establish whether this rescue extends beyond aggregate load. This would also help clarify whether the non-canonical, phosphorylation- and mitochondria-independent TDP-43 aggregation phenotype induced by PONPC recapitulates the loss-of-function pathology characteristic of ALS-associated TDP-43 proteinopathy or represents a mechanistically distinct process.

This strategy was then tested in the sALS CSF-transfer mouse model,^10^ which develops a relatively mild phenotype following exposure to sALS patient CSF. Preventive intrathecal administration of AAV5.2-PC-OxPL-VecTab blocked PC-OxPL accumulation and motor deficits, prevented MN loss and tended to normalize the inflammatory markers. Despite these encouraging results, the CSF-transfer model carries an important translational limitation. As noted by its original authors,^10^ disability and cell death remain similar across time points after CSF injection, indicating an acute, irreversible neurological insult rather than the progressive degeneration characteristic of ALS. Thus, the current AAV5.2-PC-OxPL-VecTab treatment demonstrates a preventive proof of concept rather than a test of symptomatic treatment, which would be more clinically relevant given that most patients are diagnosed after symptom onset. Treating animals after CSF infusion would help determine whether effects beyond MN protection can still provide functional benefit.

To test whether PC-OxPL specifically contributes to sALS CSF toxicity, the authors preincubated the CSF with purified PC-OxPL-VecTab protein before intrathecal injection. This *ex vivo* neutralization prevented PC-OxPL accumulation and motor deficits, further supporting a direct role for PC-OxPL in CSF toxicity. However, it does not fully establish specificity or causality, since the scFv could potentially interact with related lipid species. Fractionation, selective depletion, or reconstitution experiments—removing PC-OxPL from CSF to confirm loss of toxicity or adding purified PC-OxPL to naive CSF to determine whether it reproduces the phenotype—would offer a more definitive test of specificity. Finally, a single intrathecal dose of AAV5.2-PC-OxPL-VecTab achieved robust CNS transduction in WT minipigs, including large layer V pyramidal neurons in the motor cortex and ventral horn MNs, supporting the translational potential of this strategy. However, protein levels in CSF or plasma were not assessed. Establishing a CSF-transfer paradigm in minipigs could further strengthen the translational relevance of these findings.

Taken together, this study identifies PC-OxPL accumulation in ALS patients’ CNS; implicates ApoE as its principal carrier in CSF and shows that PC-OxPL induces ALS-relevant phenotypes that can be reversed by PC-OxPL-VecTab (Figure 1). These findings support PC-OxPL as a potential therapeutic target in ALS and provide a foundation for further mechanistic and translational studies. The critical next step will be to determine whether targeting PC-OxPL can translate into meaningful therapeutic benefit in ALS patients.

**Figure 1:**
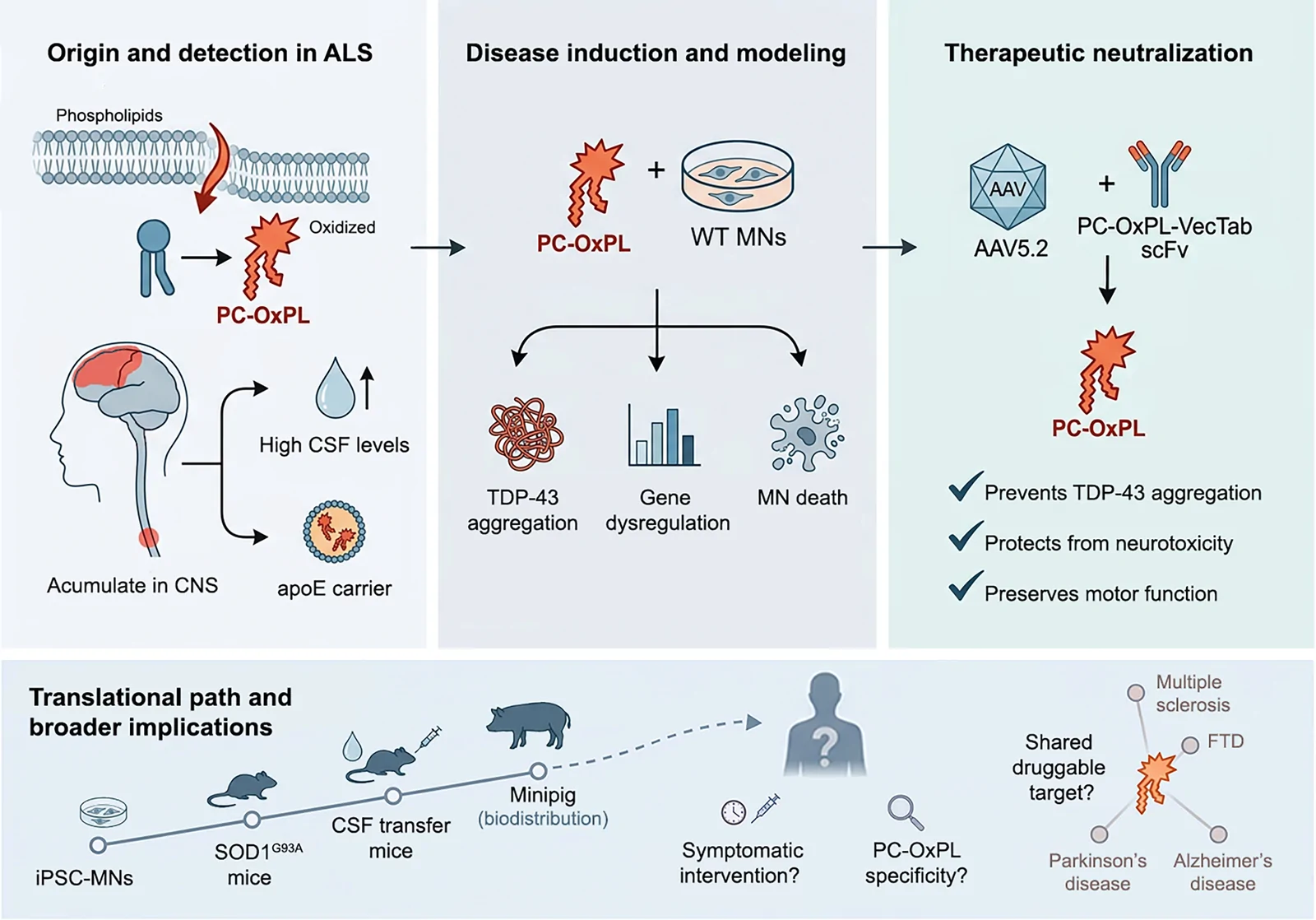
Oxidized phosphatidylcholines in ALS pathogenesis: from disease modeling to AAV-mediated therapeutic neutralization Schematic overview of the findings reported by Gomes-Duarte et al. and their translational implications. Oxidative modification of membrane phospholipids generates oxidized phosphatidylcholines (PC-OxPL), which accumulate in the CNS of ALS patients, particularly in spinal MNs, and are elevated in CSF, predominantly associated with ApoE-containing particles. Exposure of wild-type (WT) induced pluripotent stem cell-derived MNs (iPSC-MNs) to PC-OxPL induces ALS-relevant pathological features, including TDP-43 aggregation, transcriptional dysregulation, and neurotoxicity. Therapeutic neutralization using AAV5.2-delivered PC-OxPL-VecTab, a secreted single-chain variable fragment (scFv) targeting PC-OxPL, prevents TDP-43 aggregation and neurotoxicity *in vitro*, and protects against MN loss and motor deficits in a sporadic ALS CSF-transfer mouse model. Preclinical studies involving iPSC-MNs, SOD1^G93A^ mice, CSF-transfer mice, and minipigs support further therapeutic development. However, important questions remain regarding efficacy following symptom onset, target specificity, and clinical translation. The potential applicability of PC-OxPL neutralization to other neurodegenerative diseases also warrants further investigation. FTD, frontotemporal dementia.

## Declaration of interests

S.V. and A.B. report are inventors of a patent on ALS treatment based on Klotho administration to muscle tissues that is licensed to Klotho Neurosciences. A.B. is a scientific advisor of Klotho Neurosciences.

## Declaration of generative AI and AI-assisted technologies in the manuscript preparation process

The figure was created by the authors using AI-generated icons (adcreative.ai).
